# Prognosis value of lymphovascular invasion in patients with invasive ductal breast carcinoma according to lymph node metastasis status

**DOI:** 10.3332/ecancer.2022.1364

**Published:** 2022-03-03

**Authors:** Felipe Andrés Cordero da Luz, Eduarda da Costa Marinho, Camila Piqui Nascimento, Lara de Andrade Marques, Patrícia Ferreira Ribeiro Delfino, Rafael Mathias Antonioli, Rogério Agenor de Araújo, Marcelo José Barbosa Silva

**Affiliations:** 1 Center for Cancer Prevention and Research, Uberlandia Cancer Hospital, Av Amazonas nº 1996, Umuarama, Uberlândia, Minas Gerais, MG 38405-302, Brazil; 2 Laboratory of Tumor Biomarkers and Osteoimmunology, Institute of Biomedical Sciences, Federal University of Uberlandia, Av Pará nº 1720, Bloco 6T, room 07, Umuarama, Uberlândia, Minas Gerais, MG 38405-320, Brazil; 3 Faculty of Medicine, Federal University of Uberlandia, Av Pará nº 1720, Bloco 6T2u, room 07, Umuarama, Uberlândia, Minas Gerais, MG 38400-902, Brazil; a https://orcid.org/0000-0002-9381-4913; b https://orcid.org/0000-0002-1307-9104; c https://orcid.org/0000-0002-0955-8559; d https://orcid.org/0000-0002-2734-8352; e https://orcid.org/0000-0002-2196-9318; f https://orcid.org/0000-0003-3886-1562; g https://orcid.org/0000-0003-4653-6786; h https://orcid.org/0000-0002-5807-4286

**Keywords:** breast neoplasms, lymphatic metastasis, neoplasm invasiveness, neoplasm metastasis, prognosis

## Abstract

**Background:**

Tumour lymphovascular invasion is not routinely assessed in all pathology services, and whether reporting it quantitatively or qualitatively is the main factor associated with the loss of this prognostic event. This study aimed to analyse the prognostic value of qualitatively reported lymphovascular invasion in patients with invasive breast ductal carcinoma.

**Methods:**

This was a retrospective, single-center study, enrolling a total of 426 patients with invasive ductal carcinoma of the breast with a report of lymphovascular invasion, with a median follow-up of approximately 4.5 years. Kaplan–Meier and Cox regression was performed to obtain the predictive value of lymphovascular invasion. Propensity score matching was performed to reduce bias by standardising factors with significant differential distribution of lymphovascular invasion status.

**Results:**

Lymphovascular invasion was present in 197 (49.2%) patients. Multivariate Cox regression showed that lymphovascular invasion independently increases the risk of death by almost two times (adjusted hazard ratio (HR): 2.045 (1.226–3.406), *p* = 0.006) and the risk of distant metastasis by more than two times (adjusted HR: 2.373 (1.404–4.010), *p* = 0.001). Subgroup analysis after matching by propensity score in adjuvant-only patients showed that the lymphovascular invasion is a factor of increased death in N− patients (adjusted HR: 12.597 (1.624–97.728), *p* = 0.015) and of distant metastasis-free survival in N+ patients (adjusted HR: 4.862 (1.649–14.335), *p* = 0.004) and almost for N− patients (adjusted HR 7.905 (0.969–64.509), *p* = 0.004).

**Conclusion:**

The presence of lymphovascular invasion is a predictor of worse prognosis in patients with invasive ductal carcinoma of the breast, even with metastatic lymph node disease (N1–N3).

## Background

Breast cancer is the main cause of cancer deaths among women worldwide [1], and almost all deaths are due to the development of distant metastases [2]. Among several clinicopathological factors, tumour size and mainly lymph node metastasis are the main risk factors for developing secondary lesions, that is, distant metastases [3]. Although traditional breast cancer development is conceived as a linear and stepwise progression (tumour growth, lymph node metastasis and distant organ metastasis) [4], distant metastasis arises primarily from the tumoural invasion of blood or lymphatic vessels by cancer cells, known as vascular invasion [5]. Thus, the correct identification of vessel invasion is important because it increases the recurrence risk, mainly at a distance [6].

According to the St Gallen Consensus, the extensive vascular invasion is classified as a risk factor for distant recurrences [6–8]. However, this criterion can generate subjectivity and excludes the potential prognostic role of non-extensive vascular invasion [8, 9], hindering a consensus about how to report vascular invasion and if the simple qualitative report has a prognostic value [9]. Additionally, artifacts can lead to misinterpretation [9], and morphological similarities between blood and lymphatic vessels in simple histological, without costly techniques (immunohistochemistry), difficult proper analysis [10], forcing to a simple report of lymphovascular/vascular invasion, that is, invasion of non-specific vessels [5]. These difficulties are the main reasons why many laboratories avoid analysing and reporting vessel invasion in exchange for the loss of important prognostic factors.

This study retrospectively analysed the validity of simple qualitative lymphovascular report as a prognostic factor in breast cancer patients, mainly in matched patients by propensity score.

## Methods

### Study design

Retrospective longitudinal study based on data from medical records of patients with breast cancer treated in the oncology sector of the Clinical Hospital of the Federal University of Uberlandia between January 1999 and December 2019.

### Classifications and outcomes

Based on the anatomopathological examination results and not on the medical notes, all patients were reclassified in their pathological TNM according to the Seventh Edition of the American Joint Committee on Cancer (AJCC) [11]. The highest T and N between the clinical and the pathological staging in patients who underwent neoadjuvant therapy was considered for predictive analysis.

Tumours were classified as human epidermal receptor 2 + (HER2+) regardless of hormone receptor expression [estrogen receptor (ER) and/or progesterone receptor (PR)] as long as they had a 3+ score by immunohistochemistry (IHC) or *ERBB2* amplification by *in situ* hybridisation method [12].

Tumours were classified as luminal when there was a lack of in the absence of HER2 superexpression/amplification, and at least 1% of tumour cells expressed either hormone receptors (ER and/or PR) [12]. Tumour classification as Luminal A or Luminal B was based on the ideal Ki-67 cutoff established by the prognostic value within this group of patients, then being classified as >12%, which have 100% agreement of previously accepted cutoff of ≥14% [13].

Patients’ age was classified into a two-level categorization (<70 years and ≥70 years) for overall survival (OS) analysis due to shorter life expectancy, a difference in all-cause death, and intensity of treatments [14, 15].

The risk assessment was considered based on the time from anatomopathological confirmation of malignancy until analysed outcomes (death and distant metastasis).

The systemic and radiotherapy treatments were considered adequate whenever patients received treatment according to indications by current guidelines [7, 13, 16, 17]. Patients were classified as having received or not having received treatment if there was correct adherence or not to the treatment protocols, respectively, as in a previous study [18].

### Histological and IHC methods

According to the standard guidelines of the Pathology laboratory at the cited Institution, immunological and histological analyses were performed by one pathologist and independently confirmed by another pathologist.

Immunohistochemical data were retrospectively retrieved from IHC reports. IHCs were prospectively performed at the local laboratory of the Institution according to good practice guidelines preserved through time. Detection and revealing were performed by an avidin-biotin-peroxidase system.

Lymphovascular invasion was evaluated by H&E stain of histological slides and was considered positive in the presence of tumour cells inside vessels.

### Ethical aspects

This study was approved by the Human Research Ethics Committee (protocol number 803.826/14) of the local Institution and followed all the ethical principles of the Declaration of Helsinki and its subsequent amendments or comparable ethical standards. The informed consent form was waived, according to the type of study performed.

### Eligibility

Were enrolled female patients with non-metastatic invasive ductal carcinoma of no special type histology. Patients were excluded if had: incomplete immunohistochemistry, anatomopathological and clinical data; bilateral cancer; more than one primary cancer; unreported cause of death; and follow-up time less than 180 days (6 months) from diagnostic to event/censoring to avoid non-diagnosed synchronous metastasis.

From a total of 2,186, 426 were selected in this condition, with a complete clinicopathological report.

### Statistical analysis

Distributions were analysed using the Kolmogorov–Smirnov test. Continuous variables with normal distribution were described as mean (±standard deviation), and non-parametric variables as median (minimum–maximum); categorical variables were described as frequencies.

Survival analyses were performed with the Kaplan–Meier (KM) estimator using the Log-Rank test. Variables with survival proportionality by KM analysis were included in the Cox regression model to determine independent prognosis factors. Time-dependent Cox regression models were performed including the T_COV variable with interaction of the time of inflection observed in KM curves (T_COV*). The optimal Cox regression model was obtained by the Stepwise Forward Wald method, performed with an entry *p*-value equal to 0.25 and output *p*-value equal to 0.05 to reduce covariate collapsibility [19] and overfitted models.

Patients were matched by propensity score by significant variables (*p* < 0.05) like age (categorical), T, N, histological grade, molecular subtype, and neoadjuvant chemotherapy, grouping by lymphovascular invasion.

A *p*-value of <0.05 was considered to be significant. Statistical analysis was performed with IBM Statistical Package for the Social Sciences v25.0.

## Results

One hundred and ninety-seven (46.2%) patients reported the presence of lymphovascular invasion. All relevant clinical and pathological characteristics of patients are described in Table 1.

**Figure 1.**
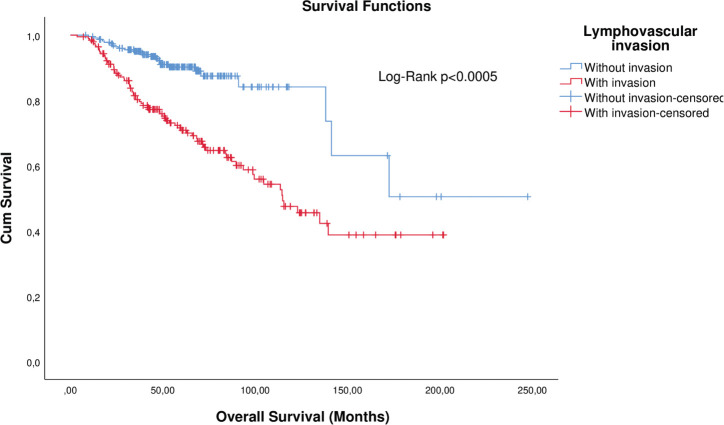
KM plot of OS considering the lymphovascular invasion in patients with breast cancer.

**Figure 2.**
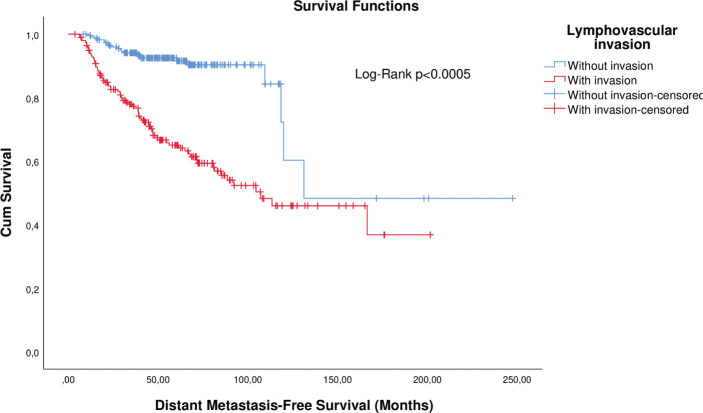
KM plot of DMFS considering the lymphovascular invasion in patients with breast cancer.

KM curve analysis showed proportionality of risks for both OS (Figure 1) and distant metastasis-free survival (DMFS) (Figure 2). By univariate Cox regression, lymphovascular invasion was observed as a prognosis factor for both OS (HR: 4.026 (2.608–6.781), *p* < 0.0005) and DMFS (HR: 3.083 (1.947–4.883), *p* = 0.006). Correction by other covariables (multivariate Cox regression) showed that lymphovascular invasion as an independent prognostic factor for both OS (HR: 2.045 (1.226–3.409), *p* = 0.006) and DMFS (HR: 2.373 (1.404–4.010), *p* = 0.001).

It was possible to observe an increasing frequency of lymphovascular invasion according to categorical classification: 21.8% (*n* = 44) of the N0, 60.2% (*n* = 80) of the N1, 76.6% (*n* = 49) of N2 and 88.9% (*n* = 24) of N3. Additionally, lymphovascular invasion is thought to be the predecessor event of lymph node metastasis [20, 21]. Due to these data, the retrospective nature of the study and the decrease of HR values of lymphovascular invasion after covariation, it is highly likely that the effect of lymphovascular invasion is influenced by lymph node metastasis and the Cox model does not eliminate all confounding. To reduce confounding, patients were matched by propensity score.

**Figure 3.**
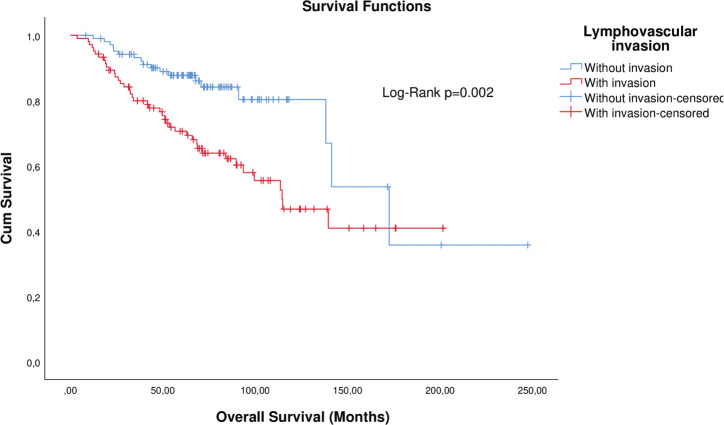
KM plot of OS considering the lymphovascular invasion in patients with breast cancer after matching.

**Figure 4.**
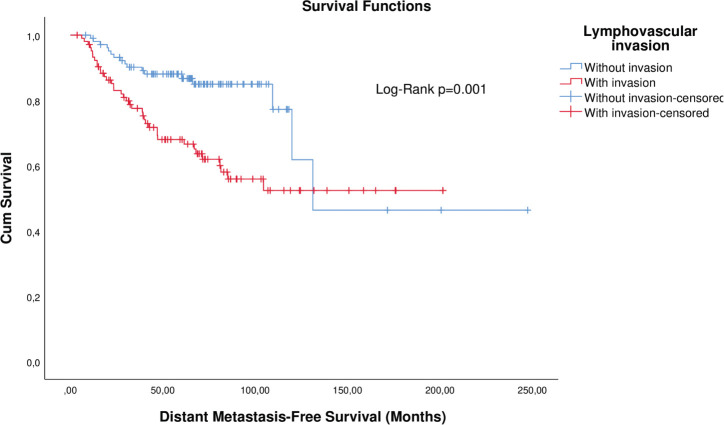
KM plot of DMFS considering the lymphovascular invasion in patients with breast cancer after matching.

Matched analysis by T, N, and neoadjuvant chemotherapy corroborated the first findings (Table 2). KM curves of matched patients are depicted in Figures 3 and 4.

To analyse any possible risk modification of lymphovascular invasion by N status, initial patients were again matched by propensity score, but first, they were separated into two groups according to the quantitative classification of N (N− and N+); patients treated with neoadjuvant chemotherapy were excluded in these analyses due to potential risk modification.

Patients with N− disease were matched by T and histological grade; patients with N+ disease were matched by T, N and histological grade. Lymphovascular invasion was shown as an independent factor for OS and a trend for distant metastasis in N− patients (Table 3), while it was significant and the sole prognostic factor for distant metastasis in N+ patients (Table 4).

Finally, it was tested whether treatment could impact in the prognosis value of lymphovascular invasion. Although the vast majority of patients who did not receive adequate treatment were due to contraindication, this may result in a modification of the prognostic factor of lymphovascular invasion.

As the analyses before pairing by propensity score showed sufficient ability to correct confounding, all adjuvant regimen patients were included in these analyses. To eliminate immortal time bias, the time from completion of adjuvant therapy, except endocrine therapy, to outcomes was considered; patients who developed metastasis during treatment were excluded, totalling 304 patients in this set of analyses. Cox regression showed that lymphovascular invasion is still and independent prognosis factor for both OS and DMFS after adjusting by treatments (Table 5).

## Discussion

The lymphovascular invasion is a long-term known prognosis factor of poorer outcome in breast cancer patients irrespective of lymph node metastasis status [20]. However, little emphasis has been given to this important risk factor due to a series of controversies and difficulty in establishing an analysis standard to determine the intensity of this phenomenon.

One of the first assessments of the prognostic value of this factor according to its intensity was by Colleoni *et al* [8], who observed that extensive but not focal or moderate invasion is a risk factor in patients with pT1-3/N0 disease. However, this classification involves both the number of tumour cells per focus and the number of foci in different paraffin tumour blocks, which implies subjectivity and the complexation of the process. Even so, this classification became the basis for risk classification by the St Gallen consensus also in 2007 [6].

The main paradigm of cancer evolution proposes a step-by-step relationship in the growth of the primary tumour, invasion of regional lymph nodes and subsequent development of a secondary tumour via metastasis. However, this relationship is not linear, and patients with small tumours and without lymph node metastasis can develop distant metastasis as well [4]. Apart from being the predecessor of lymph node metastasis [20, 21], the lymphovascular invasion is a marker of metastatic potential. Increased expression and signalling of chemokines path ways, especially CXCR4, are observed in the lymphovascular invasion of Paget’s disease of the breast [22]. While the CCR7 axis is associated with a migratory phenotype to lymphoid tissue, like regional lymph nodes, the CXCR4 axis is associated with both migrations to lymph nodes and distant organs [23]. Interestingly, Venet *et al* [24] observed that only 25% of distant metastasis share a common origin with metastatic lymph nodes in breast cancer patients. Although they observed a poorer prognosis in patients whose secondary tumours share a common origin, or are seeded by, metastatic lymph nodes, the vast majority of secondary tumours are seeded directly by the primary tumour. These data suggest that metastatic cells on the lymph node do not necessarily disseminate to distant organs, but are markers of increased metastatic potential.

Therefore, the identification of metastatic potential due to the presence of lymphovascular invasion, regardless of its extension, may have a prognostic value. In this study, differently from reported for only extensive vascular invasion, we observed that qualitative lymphovascular invasion is an important prognostic factor in breast cancer patients with either N− or N+ disease.

The results obtained by us are in fact in agreement with some studies. It was previously identified that the qualitative lymphovascular invasion, independently of its extension, is a worse prognostic factor in patients with N− disease [25–27], making it comparable to the N1 classification [27], and in patients with N+ disease [28, 29] or high-risk classifications, that are N+ as well [9].

As there is an important correlation between lymphovascular invasion and lymph node metastasis and between lymphovascular invasion and tumour size [29–31], in this study we analysed whether the lymphovascular invasion is an independent risk factor of death and distant metastasis in breast cancer patients balanced for potential confounding pathological factors. Analyses after matching by propensity score corroborated our first findings in the entire group and in subgroup analysis by N status. This is in agreement with a recent report. During the review of this manuscript, Houvenaeghel *et al* [32] published the results of a similar study involving more than 17,000 patients, obtaining similar results even after pairing. Interestingly, they also observed an important prognostic value even covariate with the administration of adjuvant therapy, except for Luminal A patients. In this regard, we observed that lymphovascular invasion persists as an independent prognostic factor in a similar way to lymph node metastasis.

These results as serious implications in therapy decisions. The former therapy decision by the St Gallen consensus is based on risk factors, and only extensive vascular invasion is considered as a factor of classification as intermediate risk [6]. For example, in patients with luminal tumours, this implies in similar risk classification of pN0 disease as pN1 disease [6, 7]. However, and chemotherapy is mandatory only in patients with pN2-N3 disease [6, 7]. Nonetheless, we observed that the presence of lymphovascular invasion is a risk factor even in N+ patients. Fortunately, in the 16th St Gallen consensus, which took place in 2019, it was established that the inclusion of chemotherapy for N0 and N1 patients will also be based on the presence of lymphovascular invasion, despite not having specified which extension of the invasion is used in this new criterion [33]. However, the identification of lymphovascular invasion is not yet widely incorporated in many pathology laboratories. For example, in Brazil, its assessment is not part of the diagnostic guidelines used in the public treatment network [34].

This study has some limitations. The main limitation is the elapsed time, which involved the incorporation and change of therapies schemas. Although this is something to be expected over time and we have classified the treatment according to more recent guidelines [6, 7, 13, 35], these changes make it difficult to draw concrete conclusions regarding the prognostic value of lymphovascular invasion.

Another limitation is the stratification of patients for further analysis. While some note that the lymphovascular invasion as prognosis factor only applicable to in patients with N0 disease with tumours up to 5 cm (pT2) [27] or up to 2 cm (pT1) [36], or is modified by other risk factors [9], or have distinct prognosis factor according to the tumour subtypes [32], we face limitations due to the small number of patients included in this study. However, our global analyses are in agreement with results of also global analyses of other studies, such as from Houvenaeghel *et al* [32].

Another possible limitation is the type of vessel involved in this phenomenon. Even though other studies are also based on simple histology analyses of slides stained with haematoxylin and eosin [9, 32], invasion of blood vessels has a prognostic value independent of lymphovascular invasion [37]. Although the first one is rarer than the second [37, 10], different migration mechanisms may be involved, as mentioned above, which could help to refine the identification of patients at higher risk of distant metastases and therapeutic benefit.

A larger study analysing both blood and lymphatic vessel invasion, either separately or in combination, is necessary to corroborate the predictive value of lymphovascular invasion report by common histological analysis in breast cancer patients.

## Conclusion

The presence of lymphovascular invasion is a predictor of worse prognosis in breast cancer patients, either N− and N+, and must be routinely assessed and must be reported even in qualitative terms (absence/presence) in pathology services.

## Conflicts of interest

The authors declared no conflict of interests.

## Funding

The authors received no financial support for the research, authorship and/or publication of this article.

## Authors’ contributions

FACL conceived the study. FACL, ECM, CPN, LAM, PFRD designed methodology and collected data. FACL performed data analysis. RMF, RAR and MJBS supervised and validated data collection and analysis. FACL wrote initial manuscript. All authors reviewed and approved final draft.

## Figures and Tables

**Table 1.** Clinical data of included patients (*n* = 426).

Variable	*N* (%)	Median (min.–max.)
Time of observation	426 (100%)	55.45 months (3.67–247.40)
Time to distant relapse	426 (100%)	52.60 months (3.67–247.40)
Age	426 (100%)	56 years (26–92)
Age		
<70 years	362 (85.0%)	
≥70 years	64 (15.0%)	
Deaths		
No	329 (77.2%)	
Yes	97 (22.8%)	
Distant relapse		
No	329 (77.2%)	
Yes	97 (22.8%)	
Surgery		
Breast-conserving	200 (46.9%)	
Mastectomy	226 (53.1%)	
Surgical margin		
Negative	384 (90.1%)	
Positive	42 (9.9%)	
T		
T1	111 (26.1%)	
T2	187 (43.9%)	
T3	78 (18.3%)	
T4	50 (11.7%)	
N		
N0	202 (47.4%)	
N1	133 (31.2%)	
N2	64 (15.0%)	
N3	27 (6.3%)	
Stage		
I	81 (19.0%)	
IIA	114 (26.8%)	
IIB	88 (20.7%)	
III	143 (33.6%)	
Lymphovascular invasion		
Absent	229 (53.8%)	
Present	197 (46.2%)	
Molecular subtype		
Luminal A	116 (27.2%)	
Luminal B	160 (37.6%)	
HER2	76 (17.8%)	
Triple-negative	74 (17.4%)	
Estrogen receptor		
Negative	120 (28.2%)	
Positive	306 (71.8%)	
Progesterone receptor		
Negative	162 (38.0%)	
Positive	264 (62.0%)	
Hormone receptor (any)		
Negative	107 (25.1%)	
Positive	319 (74.9%)	
Histological grade		
G1	48 (11.3%)	
G2	269 (63.1%)	
G3	109 (25.6%)	
Chemotherapy		
No	77 (18.1%)	
Neoadjuvant	119 (27.9%)	
Adjuvant	230 (54.0%)	
Endocrine therapy (*n* = 319)		
No	11/319 (3.4%)	
Yes	308/319 (96.6%)	
Trastuzumab (*n* = 76)		
No	30/76 (39.5%)	
Yes	46/76 (60.5%)	
Systemic treatment		
No	67 (15.7%)	
Yes	359 (84.3%)	
Radiotherapy		
No	31 (7.3%)	
Yes	395 (92.7%)	

G1 – well differentiated; G2 – moderately differentiated; G3 – poorly differentiated; N – lymph node metastasis; T – tumour size

**Table 2.** Univariate and multivariate time-dependent Cox regression analyses for OS and metastasis-free survival according to risk factors in all patients after matching by T, N and neoadjuvant chemotherapy (*n* = 210).

OS				
	Univariate		Multivariate	
Factor	HR (95% CI)	*p*	HR (95% CI)	*p*
Lymphovascular invasion				
Absent	1		1	
Present	2.411 (1.379–4.214)	0.002	2.292 (1.307–4.023)	0.004
T				
T1	1			
T2	1.681 (0.649–4.354)	0.285		
T3	2.468 (0.893–6.818)	0.082		
T4	2.559 (0.865–7.566)	0.089		
N				
N0	1		1	
N1	1.397 (0.765–2.552)	0.276	1.404 (0.766–2.572)	0.273
N2	1.949 (0.930–4.087)	0.077	2.960 (1.377–6.360)	0.005
N3	11.314 (3.125–40.970)	<0.0005	6.798 (1.827–25.301)	0.004
Histological grade				
G1/G2	1		1	
G3	2.431 (1.431–4.130)	0.001	2.085 (1.189–3.656)	0.010
Molecular subtype				
Luminal A	1		1	
Luminal B	1.675 (0.762–3.681)	0.199	1.193 (0.530–2.685)	0.671
HER2	1.083 (0.417–2.816)	0.870	0.680 (0.247–1.871)	0.455
Triple-negative	4.718 (2.165–10.281)	<0.0005	2.085 (1.189–3.656)	0.010
DMFS				
Lymphovascular invasion				
Absent	1		1	
Present	2.584 (1.455–4.588)	0.001	2.305 (1.294–4.103)	0.005
T				
T1	1			
T2	1.825 (0.691–4.823)	0.225		
T3	2.795 (1.007–7.755)	0.048		
T4	3.359 (1.135–9.942)	0.029		
N				
N0	1		1	
N1	2.279 (1.183–4.387)	0.014	2.267 (1.170–4.393)	0.015
N2	3.076 (1.373–6.892)	0.006	3.377 (1.477–7.725)	0.004
N3	3.267 (0.716–14.912)	0.127	3.139 (0.655–15.033)	0.152
Histological grade				
G1/G2	1		1	
G3	2.155 (1.252–3.709)	0.006	1.738 (0.986–3.061)	0.056
Molecular subtype				
Luminal A	1		1	
Luminal B	1.927 (0.805–4.614)	0.141	1.451 (0.591–3.565)	0.417
HER2	2.201 (0.853–5.680)	0.103	1.603 (0.599–4.294)	0.347
Triple-negative	5.105 (2.140–12.182)	<0.0005	4.093 (1.674–10.088)	0.002

G1 – well differentiated; G2 – moderately differentiated; G3 – poorly differentiated; N – lymph node metastasis; T – tumour size

**Table 3.** Univariate and multivariate Cox regression analyses for outcomes in N− adjuvant patients after matching (*n* = 78).

OS				
	Univariate		Multivariate	
Factor	HR (95% CI)	*p*	HR (95% CI)	*p*
Lymphovascular invasion				
Absent	1		1	
Present	12.597 (1.624–97.728)	0.015	12.597 (1.624–97.728)	0.015
T				
T1	1			
T2	3.418 (0.440–26.541)	0.240		
T3	2.197 (0.137–35.309)	0.579		
Histological grade				
G1/G2	1			
G3	2.374 (0.774–7.276)	0.130		
Molecular subtype				
Luminal A	1			
Luminal B	1.784 (0.327–9.748)	0.504		
HER2	1.958 (0.274–13.965)	0.503		
Triple-negative	4.398 (0.846–22.868)	0.078		
DMFS				
Lymphovascular invasion				
Absent	1		1	
Present	7.905 (0.969–64.509)	0.054	7.905 (0.969–64.509)	0.054
T				
T1	1			
T2	Undefined	0.955		
T3	Undefined	0.955		
Histological grade				
G1/G2	1			
G3	3.812 (0.948–15.326)	0.059		
Molecular subtype				
Luminal A	1			
Luminal B	Undefined	0.950		
HER2	Undefined	0.949		
Triple-negative	Undefined	0.947		

G1 – well differentiated; G2 – moderately differentiated; G3 – poorly differentiated; T – tumour size

**Table 4.** Univariate and multivariate Cox regression analyses for outcomes in N+ adjuvant patients after matching (*n* = 66).

OS				
	Univariate		Multivariate	
Factor	HR (95% CI)	*p*	HR (95% CI)	*p*
Lymphovascular invasion				
Absent	1			
Present	2.139 (0.741–6.174)	0.160		
T				
T1	1			
T2	2.461 (0.836–7.244)	0.102		
T3	2.653 (0.332–20.935)	0.359		
N				
N1	1			
N2	2.461 (0.836–7.244)	0.102		
N3	2.635 (0.332–20.935)	0.359		
Histological grade				
G1/G2	1		1	
G3	4.721 (1.534–14.523)	0.007	4.721 (1.534–14.523)	0.007
Molecular subtype				
Luminal A	1			
Luminal B	9.676 (1.236–75.773)	0.031		
HER2	4.154 (0.375–46.002)	0.246		
Triple-negative	10.333 (1.144–93.360)	0.038		
DMFS				
Lymphovascular invasion				
Absent	1		1	
Present	3.299 (1.196–9.102)	0.021	4.862 (1.649–14.335)	0.004
T				
T1	1			
T2	1.896 (0.553–6.498)	0.309		
T3	Undefined	0.981		
N				
N1	1			
N2	1.800 (0.647–5.010)	0.260		
N3	1.494 (0.191–11.666)	0.702		
Histological grade				
G1/G2	1			
G3	2.090 (0.670–6.525)	0.204		
Molecular subtype				
Luminal A	1		1	
Luminal B	5.355 (1.169–24.533)	0.031	8.008 (1.675–38.294)	0.009
HER2	4.445 (0.813–24.313)	0.085	3.862 (0.704–21.203)	0.120
Triple-negative	5.770 (1.052–31.648)	0.044	8.572 (1.530–48.028)	0.015

G1 – well differentiated; G2 – moderately differentiated; G3 – poorly differentiated; N – lymph node metastasis

**Table 5.** Univariate and multivariate Cox regression analyses for OS and metastasis-free survival according to risk factors and treatments in patients of adjuvant schema (*n* = 304).

OS				
	Univariate		Multivariate	
Factor	HR (95% CI)	*p*	HR (95% CI)	*p*
Lymphovascular invasion				
Absent	1		1	
Present	3.597 (1.878–6.890)	0.002	2.656 (1.331–5.299)	0.006
T				
T1	1		1	
T2	2.865 (1.378–5.957)	0.005	2.543 (1.197–5.405)	0.015
T3	1.281 (0.346–4.749)	0.711	0.590 (0.149–2.335)	0.453
T4	10.653 (3.536–32.093)	<0.005	6.513 (1.972–21.513)	0.002
N				
N0/N1	1		1	
N2/N3	3.316 (1.885–5.833)	<0.0005	1.995 (1.052–3.782)	0.034
Histological grade				
G1/G2	1		1	
G3	2.177 (1.191–3.980)	0.011	1.868 (0.955–3.652)	0.068
Molecular subtype				
Luminal A	1		1	
Luminal B	2.679 (1.249–5.748)	0.011	2.591 (1.177–5.706)	0.018
HER2	2.452 (0.971–6.196)	0.058	1.134 (0.374–3.433)	0.825
Triple-negative	3.045 (1.236–7.502)	0.015	4.029 (1.519–10.688)	0.005
Systemic treatment				
No	1		1	
Yes	0.558 (0.308–1.013)	0.055	0.532 (0.238–1.190)	0.124
Radiotherapy				
No	1		1	
Yes	0.915 (0.329–2.542)	0.864	1.222 (0.401–3.721)	0.724
DMFS				
Lymphovascular invasion				
Absent	1		1	
Present	4.369 (2.236–8.535)	0.001	2.815 (1.333–5.941)	0.007
T				
T1	1			
T2	2.297 (1.163–4.539)	0.017		
T3	1.106 (0.308–3.975)	0.877		
T4	6.364 (2.014–20.110)	0.002		
N				
N0	1		1	
N1	3.030 (1.470–6.247)	0.003	2.200 (1.019–4.749)	0.045
N2/N3	7.720 (3.787–15.737)	<0.0005	4.451 (2.001–9.904)	0.004
Histological grade				
G1/G2	1			
G3	1.525 (0.797–2.919)	0.203		
Molecular subtype				
Luminal A	1		1	
Luminal B	2.316 (1.106–4.849)	0.026	2.589 (1.195–5.608)	0.016
HER2	2.391 (0.993–5.760)	0.052	1.069 (0.388–2.943)	0.897
Triple-negative	2.118 (0.835–5.373)	0.114	2.996 (1.160–7.739)	0.023
Systemic treatment				
No	1		1	
Yes	0.456 (0.253–0.822)	0.009	0.430 (0.198–0.936)	0.033
Radiotherapy				
No	1		1	
Yes	0.536 (0.212–1.355)	0.188	0.407 (0.155–1.071)	0.069

G1 – well differentiated; G2 – moderately differentiated; G3 – poorly differentiated; N – lymph node metastasis; T – tumour size
